# The chlamydia knowledge, awareness and testing practices of Australian general practitioners and practice nurses: survey findings from the Australian Chlamydia Control Effectiveness Pilot (ACCEPt)

**DOI:** 10.1186/1471-2296-14-169

**Published:** 2013-11-13

**Authors:** Rebecca Lorch, Jane Hocking, Meredith Temple-Smith, Matthew Law, Anna Yeung, Anna Wood, Alaina Vaisey, Basil Donovan, Christopher K Fairley, John Kaldor, Rebecca Guy

**Affiliations:** 1 The Kirby Institute, University of New South Wales, Sydney, NSW, Australia; 2 Centre for Women’s Health, Gender and Society, Melbourne School of Population Health, University of Melbourne, Melbourne, Victoria, Australia; 3 Department of General Practice, University of Melbourne, Melbourne, Victoria, Australia; 4 Melbourne Sexual Health Centre, Carlton, Victoria, Australia

## Abstract

**Background:**

ACCEPt, a large cluster randomized control trial, aims to determine if annual testing for 16 to 29 year olds in general practice can reduce chlamydia prevalence. ACCEPt is the first trial investigating the potential role of practice nurses (PN) in chlamydia testing. To inform the design of the ACCEPt intervention, we aimed to determine the chlamydia knowledge, attitudes, and testing practices of participating general practitioners (GPs) and PNs.

**Methods:**

GPs and PNs from 143 clinics recruited from 52 areas in 4 Australian states were asked to complete a survey at time of recruitment. Responses of PNs and GPs were compared using conditional logistic regression to account for possible intra cluster correlation within clinics.

**Results:**

Of the PNs and GPs enrolled in ACCEPt, 81% and 72% completed the questionnaire respectively. Less than a third of PNs (23%) and GPs (32%) correctly identified the two age groups with highest infection rates in women and only 16% vs 17% the correct age groups in men. More PNs than GPs would offer testing opportunistically to asymptomatic patients aged ≤25 years; women having a pap smear (84% vs 55%, P<0.01); antenatal checkup (83% vs 44%, P<0.01) and Aboriginal men with a sore throat (79% vs 33%, P<0.01), but also to patients outside of the guideline age group at the time of the survey; 26 year old males presenting for a medical check (78% vs 30%, P = <0.01) and 33 year old females presenting for a pill prescription (83% vs 55%, P<0.01). More PNs than GPs knew that retesting was recommended after chlamydia treatment (93% vs 87%, P=0.027); and the recommended timeframe was 3 months (66% vs 26%, P<0.01). A high proportion of PNs (90%) agreed that they could conduct chlamydia testing in general practice, with 79% wanting greater involvement and 89% further training.

**Conclusions:**

Our survey reveals gaps in chlamydia knowledge and management among GPs and PNs that may be contributing to low testing rates in general practice. The ACCEPt intervention is well targeted to address these and support clinicians in increasing testing rates. PNs could have a role in increasing chlamydia testing.

## Background

*Chlamydia trachomatis* (hereafter referred to as chlamydia) is the most common reportable infection in the United States (US), Europe and Australia. In 2011, around 1.4 million new chlamydia diagnoses were reported in the US, 344 491 in 24 European Union member states during 2010 and 80 800 in Australia in 2011 [1-3]. In these countries, notifications of chlamydia are increasing steadily each year, with most cases in 15-29 year olds [1-3]. Without treatment, chlamydia may persist for a year or longer, with an estimated 10% of young women developing pelvic inflammatory disease within a year [4,5]. Untreated chlamydia is also an important cause of other serious reproductive morbidity including infertility and ectopic pregnancy [6,7].

Most chlamydia infections are asymptomatic [7], and in the absence of screening they may remain undetected. Clinical guidelines in various countries recommend regular testing of sexually active women aged less than 25 years, with England extending the recommendation to include males, and Australia recently extending to males and up to age 29 years [8-10]. Australian general practice clinics are ideally placed to conduct widespread chlamydia screening with 86% of women and 64% of men aged 16-29 years of age visiting a general practitioner (GP) at least once each year; however testing rates in this age group are low at 12.1% in women and 4.8% in men [11].

The Australian Government has funded the Australian Chlamydia Control Effectiveness Pilot (ACCEPt), a cluster randomised controlled trial of annual chlamydia testing in 16-29 year old males and females. ACCEPt aims to determine whether annual chlamydia screening in general practice can reduce the prevalence of chlamydia. Clinics are randomised to either receive a multifaceted intervention designed to facilitate increased testing or to continue with usual care. Further details about the intervention are described elsewhere [12]. Given the growth of the role of the practice nurse (PNs) (employed in around 60% of Australia’s general practices) in recent years, there is an opportunity for PNs to take a role in chlamydia testing [13,14]. ACCEPt clinics randomised to the intervention arm can choose for PNs to become more involved in testing and management and receive PN specific education and financial incentives. Although PNs have been recognized as having the potential to play a key role in the provision of sexual health services in general practice in the UK [15,16] to our knowledge, ACCEPt is also the first chlamydia screening trial to formally evaluate the impact of practice nurses in increasing chlamydia testing rates.

To inform the design of the ACCEPt intervention in supporting GPs and PNs to increase testing rates and to assess the scope for PN involvement in chlamydia testing in clinics, we aimed to determine the chlamydia knowledge, attitudes, awareness and testing practices of participating GPs and PNs prior to randomization and education. Although other surveys have been conducted among GPs in Australia [17-21], none have included PNs. We also assessed if PNs demonstrated any differences in these outcomes compared with GPs which could require tailoring of the intervention.

## Methods

### Setting

In Australia, there are 7 093 general practice clinics [22]. Patients can be registered at multiple clinics and are able to consult with multiple doctors. The majority of the patient’s costs for a GP consultation are covered by Medicare Australia, the universal medical insurance scheme [23]. Clinics receive most of their income by fee per service, with ~85% directly billed to the Australian government through the Medicare rebate system. Income is also generated through patient fees charged in addition to the Medicare rebate and a small proportion through government incentive payments for activities aimed at encouraging general practices to improve patient health outcomes and quality of care [24]. In the past, GP clinics were also able to receive rebates for PNs involvement in a select range of duties such as wound dressings, immunisations and cervical screening and thus their work often focused on these income generating areas [25]. Recent restructuring of PN funding arrangements into a single funding stream, aiming to support an enhanced role for PNs, now allows them to undertake a broader range of activities in areas including preventative health [13].

There are 143 general practice clinics participating in ACCEPt, based in 52 rural areas (postcodes) of New South Wales, Victoria, Queensland and South Australia. Areas were classified as rural according to 2006 Australian Bureau of Statistics census data [26]. ACCEPt towns were selected from geographical areas within Divisions of General Practice with a minimum population size of 500 16 to 29 year olds. All clinics in the towns were eligible for participation if they were a general practice. If one or more clinics in the area declined to participate, then the area was considered ineligible for participation. An additional 9 clinics in metropolitan areas participated and were eligible if they saw a minimum of 200 patients aged 16 to 29 years each month. These metropolitan clinics were recruited to assess feasibility and did not formally participate in the trial.

### Study design

A cross sectional survey examining chlamydia knowledge, awareness and testing practices was conducted among PNs and GPs participating in ACCEPt.

#### Participants

All PNs and GPs who had provided consent to participate in ACCEPt were invited to complete the survey at time of clinic recruitment, prior to randomisation of that clinic to either the control or intervention arm of the trial and before any education was undertaken. Overall, 90% of clinics approached agreed to participate in ACCEPt and 75% of GPs from participating clinics were recruited. PNs were recruited if the clinic practice manager or principal GP thought they may have a role in chlamydia testing.

#### Questionnaire administration and reminders

Participants were encouraged to complete the survey immediately after completing the ACCEPt consent documentation at the initial clinic recruitment meeting, and hand it back to research staff. Participants who were unable to complete the form at the clinic recruitment meeting were provided with reply paid envelopes in which to return surveys. Non-responders were reminded via the practice manager of the clinic, or though a letter sent by the researchers.

#### Questionnaire content

A self administered, paper questionnaire was used. There were separate questionnaires for GPs and PNs. Both questionnaires captured clinician demographics, clinical experience, training, chlamydia knowledge and testing and management practices. A number of case vignettes that described a range of patient presentations were included and respondents indicated whether chlamydia testing should be offered and the correct specimens to be collected. The GP survey included additional questions about chlamydia treatment and the complications of chlamydia, and the PN survey had additional questions about opinions regarding chlamydia testing and perceived barriers to increasing chlamydia testing. Responses were mostly Likert scales with scores of 1 to 5. The GP survey was designed first and used as a basis for the PN survey, with questions removed, modified or added to reflect the PNs scope of practice and the variability of PN roles. We conducted pilot testing with both GPs and PNs, to ensure validity with the target group and feasibility of questionnaire length.

Correct answers for the knowledge and practice questions were based on data from the National Notifiable Diseases Surveillance System, information from the Royal Australian College of General Practitioners (RACGP) Guidelines for Preventive Activities in General Practice, current at the time of the survey and The National Management Guidelines for Sexually Transmissible Infections [9,27].

#### Data analysis

Conditional logistic regression was used to explore the differences in the responses between PNs and GPs and to account for any possible intra cluster correlation within clinics. Odds ratios (OR) and confidence intervals (CI) were obtained, and p-values of <0.05 were considered statistically significant. Descriptive statistics were used to examine the responses of GPs to questions about chlamydia treatment and the responses of PN to the questions about opinions regarding chlamydia testing and perceived barriers to increasing chlamydia testing. Analysis was carried out using Stata 12.0 (College Station, TX).

#### Ethical approval

The ACCEPt trial received ethical approval from the Royal Australian College of General Practitioners National Research and Evaluation Ethics Committee, the Aboriginal Health and Medical Research Council Ethics Committee and the University of Melbourne Human Research Ethics Committee.

## Results

### Characteristics of participants

At the time of the survey 146 PNs and 773 GPs were enrolled in ACCEPt, with 118 (81%) and 556 (72%) returning the survey, respectively. Most of the PN participants (98%) were female, compared with 39% of GP participants. The majority of the participants were aged between 30-59 years (84%); a slightly higher proportion of PNs were aged 45-59 years (51% vs 46%) compared with GPs but the difference was not significant.

The majority of participants (84%) had been qualified for longer than ten years, but PNs had worked in the field of general practice for less time than GPs. PNs were more likely than GPs to have undertaken their primary training in Australia rather than overseas (93% vs 59%) and more likely than GPs to have a special interest in sexual health (47% vs 29%) (see Table 1). At the time of the survey, under half of the PNs (41%) reported involvement in chlamydia testing, whilst most GPs (81%) indicated that they were performing between 1 - 10 chlamydia tests per month.

**Table 1:** Characteristics and chlamydia knowledge of participants

**Characteristic**	**Overall n (%)**	**GP n (%)**	**PN n (%)**	**OR (95% CI)**	**P value**
**Sex**					
Male	340 (50.4)	338 (60.8)	2 (1.7)	Ref	
Female	334 (49.6)	218 (39.2)	116 (98.3)	110.8 (15.2, 806.6)	<0.01
**Age group**					
<30 years	42 (6.2)	35 (6.3)	7 (5.9)	Ref	
30-44 years	54 (37.7)	215 (38.7)	39 (33.1)	0.9 (0.3, 2.8)	0.96
45-59 years	313 (46.4)	253 (45.5)	60 (50.9)	1.6 (0.5, 4.5)	0.36
>60 years	65 (10)	53 (10)	12 (10)	1.3 (0.4, 4.6)	0.69
**Years Qualified**					
<5 years	20 (3.0)	14 (2.5)	6 (5.2)	Ref	
5-10 years	86 (12.9)	75 (13.6)	11 (9.5)	0.8 (0.2, 2.9)	0.70
10-20 years	168 (25.2)	144 (26.2)	24 (20.7)	0.5 (0.2, 1.8)	0.33
>20 years	392 (58.9)	317 (57.6)	75 (64.7)	1.1 (0.4, 3.6)	0.83
**Years working in general practice**					
<5 years	219 (32.8)	155 (28.2)	64 (54.2)	Ref	
5-10 years	87 (13.0)	63 (11.4)	24 (19.5)	0.9 (0.5, 1.9)	0.94
10-20 years	144 (21.6)	121 (22.0)	23 (19.5)	0.5 (0.3, 0.9)	0.03
>20 years	218 (32.6)	211 (38.4)	7 (5.9)	0.1 (0.0, 0.2)	<0.01
**Country of training**					
Overseas	235 (35.1)	227 (41.0)	8 (6.9)	Ref	
Australia	435 (64.9)	327 (59.0)	108 (93.1)	18.6 (7.0, 49.3)	<0.01
**Interest in sexual health**					
No	449 (67.6)	386 (70.7)	63 (53.4)	Ref	
Yes	215 (32.4)	160 (29.3)	55 (46.6)	1.7 (1.0, 2.8)	0.05
**Questions related to chlamydia knowledge**					
**Which age groups have the highest rates of chlamydia infection in women?**					
Incorrect	451 (69.8)	363 (68.4)	88 (76.5)	Ref	
Correct*	195 (30.2)	168 (31.6)	27 (23.5)	0.6 (0.3, 1.1)	0.08
**Which age groups have the highest rates of chlamydia infection in men?**					
Incorrect	516 (83.2)	423 (83.1)	93 (83.8)	Ref	
Correct**	104 (16.8)	86 (16.9)	18 (16.2)	1.0 (0.5, 1.9)	0.94
**Most chlamydia infections are asymptomatic in women**					
Disagree	67 (10.0)	53 (9.6)	14 (12.0)	Ref	
Agree^	600 (90.0)	498 (90.4)	102 (88.0)	0.5 (0.2, 1.0)	0.06
**Most chlamydia infections are asymptomatic in men**					
Disagree	157 (23.7)	136 (24.9)	21 (18.3)	Ref	
Agree^	500 (75.5)	406 (74.2)	94 (81.7)	1.0 (0.6, 1.8)	0.98

*Correct answer = age groups 15-19 and 20-24 years *both* identified **Correct answer = age groups 20-24 and 25-29 years *both* identified ^Correct answer.

GP, general practitioner.

PN, practice nurse.

OR, odds ratio.

CI, confidence Interval.

### Chlamydia knowledge

Less than a third of PNs and GPs could correctly identify both age groups (16-19, and 20-24 years) with the highest rates of chlamydia in women (23% vs 32%), and an even lower proportion correctly identified both age groups (20-24 and 25-29 years) in men (16% vs 17%) with no significant differences between PNs and GPs. The majority of PNs and GPs correctly agreed with the statement that “most chlamydia infections are asymptomatic” in women (88% vs 90%) with a slightly lower proportion agreeing with this statement for men (82% vs 74%). There were no significant differences between the two professions (see Table 1).

### Chlamydia practice

#### Testing scenarios

Close to 80% of PNs compared with about half of the GPs, correctly identified that chlamydia testing should be offered opportunistically in the following asymptomatic clinical scenarios; a 23 year old female presenting for a pap smear (84% vs 57%), a 24 year old pregnant female (85% vs 45%) and a 22 year old Aboriginal or Torres Strait Islander male with a sore throat (79% vs 34%). Similarly, a higher proportion of PNs compared with GPs identified that testing should be conducted in two other opportunistic scenarios which at the time were outside the recommended age group; a 26 year old male presenting for a truck license medical check (78% vs 30%) and a 33 year old female presenting for a pill prescription (83% vs 55%). The difference between professions were significant (p < 0.05 for all).

Nearly all PNs and GPs (around 97%) correctly identified that testing should be offered in young people presenting with STI related symptoms (an 18 year old female with abdominal pain and a 17 year old male with genital warts) and when there is a risk of STIs (a 34 year old male wanting a HIV test) (see Table 2).

**Table 2:** Chlamydia practice - testing scenarios and specimens

**Questions relating to chlamydia practice**	**Overall n (%)**	**GP n (%)**	**PN n (%)**	**OR (95% CI)**	**P value**
**Should chlamydia testing be offered to: 23 year old female, pap smear**					
No	259 (38.6)	240 (43.3)	19 (16.4)	Ref	
Yes*	411 (61.3)	314 (56.7)	97 (83.6)	3.3 (1.8, 6.0)	<0.01
**18 year old woman, lower abdominal pain**					
No	28 (4.2)	27 (4.9)	1 (0.9)	Ref	
Yes*	642 (95.8)	528 (95.1)	114 (99.1)	5.6 (.6, 50.8)	0.12
**26 year old man, truck license medical examination**					
No*	466 (69.5)	432 (77.8)	34 (29.6)	Ref	
Yes	204 (30.5)	123 (22.2)	81 (70.4)	0.1 (0.1, 0.2)	<0.01
**24 year old woman, 16 weeks pregnant**					
No	322 (48.2)	304 (55.1)	18 (15.6)	Ref	
Yes*	345 (51.7)	248 (44.9)	97 (84.4)	7.3 (3.6, 14.6)	<0.01
**22 year Aboriginal man, sore throat**					
No	393 (58.6)	369 (66.5)	24 (20.8)	Ref	
Yes*	277 (41.3)	186 (33.5)	91 (79.1)	7.1 (3.9, 12.9)	<0.01
**33 year old woman in stable relationship, pill script**					
No*	523 (78.2)	459 (83.0)	64 (55.2)	Ref	
Yes	146 (21.8)	94 (17.0)	52 (44.8)	0.3 (0.2, 0.5)	<0.01
**17 year old man, genital warts**					
No	20 (3.0)	17 (3.1)	3 (2.6)	Ref	
Yes*	651 (97.0)	538 (96.9)	113 (97.4)	1.4 (.4, 5.4)	0.62
**34 year old man, 2 female partners in 6 months, HIV test**					
No	16 (2.4)	13 (2.4)	3 (2.6)	Ref	
Yes*	652 (97.6)	540 (97.6)	112 (97.4)	1.0 (0.2, 5.4)	0.98
**Which specimens can be used to test for chlamydia in the following patients:**					
**Heterosexual female, no genital symptoms**^**1**^					
Incorrect	29 (4.4)	14 (2.6)	15 (13.3)	Ref	
Correct	631 (95.6)	533 (97.4)	98 (86.7)	0.15 (0.1, 0.4)	<0.001
**Heterosexual female, abnormal vaginal/cervical discharge**^**2**^					
Incorrect	44 (6.6)	19 (3.5)	25 (22.1)	Ref	
Correct	620 (93.4)	532 (96.5)	88 (77.9)	0.13 (0.1, 0.3)	<0.01
**Heterosexual male, no genital symptoms**^**3**^					
Incorrect	62 (9.5)	31 (5.7)	31 (27.7)	Ref	
Correct	592 (90.5)	511 (94.3)	81 (72.3)	0.14 (0.1, 0.3)	<0.01
**Heterosexual man, urethral discharge**^**4**^					
Incorrect	502 (76.4)	404 (74.3)	98 (86.7)	Ref	
Correct	155 (23.6)	140 (25.7)	15 (13.3)	0.45 (0.2, 0.9)	0.03
**Man who has sex with men, no genital symptoms**^**5**^					
Incorrect	203 (81.2)	146 (78.9)	57 (87.7)	Ref	
Correct	47 (18.8)	39 (21.1)	8 (12.3)	0.28 (0.1, 1.0)	0.05

*Correct answer.

^1^Acceptable specimens - cervical or high vaginal or self collected vaginal swabs or urine ^2^Acceptable specimens - cervical or high vaginal or self collected vaginal swabs or urine ^3^Acceptable specimens - urine ^4^Accepatble specimens - urine ^5^Acceptable specimens - anal swab and urine.

GP, general practitioner.

PN, practice nurse.

OR, odds ratio.

CI, confidence Interval.

#### Specimen collection

Although most PNs and GPs correctly identified the appropriate specimens to be used for asymptomatic and symptomatic patient presentations in women and asymptomatic presentations in heterosexual men, PNs were less likely to report correct responses for all three presentations (see Table 2). A much lower proportion of both GPs and PNs could identify the appropriate specimens (urine) for a heterosexual male with urethral discharge (26% vs 13%, p = 0.03) with three-quarters incorrectly indicating a urethral swab should be collected, and the appropriate specimen (urine, and rectal swab) for an asymptomatic man who has sex with men (MSM) (21% vs 12%, p = 0.05) with two-thirds failing to indicate a patient self-collected rectal swab should be collected. The differences between professions were all significant (see Table 2).

#### Retesting

PNs were more likely than GPs to correctly identify that a follow up test is recommended after a negative result (74% vs 33%, OR 4.9, 95% CI 2.9, 8.6) and it should be conducted at one year (80% vs 51%, OR 3.3, 95% CI 1.1, 9.3), that a repeat test should be done after a positive chlamydia result (93% vs 87%, OR 2.7, 95% CI 1.1, 6.4), and the appropriate time frame is three months (66% vs 26%, OR 3.8, 95% CI 1.8, 8.11) (p < 0.05 for all).

#### GP chlamydia treatment practices

When asked to choose from a possible list of antibiotics that they usually prescribe for men or non- pregnant women, the majority of GPs (91%) correctly indicated that they usually prescribe azithromycin. In the case of pregnant women with uncomplicated chlamydia infection, under half (41%) would usually prescribe the recommended antibiotic (azithromycin), with 22% choosing erythromycin and 16% amoxicillin.

#### PN opinions and perceived barriers to testing

A high proportion of PNs agreed that they could conduct chlamydia testing in general practice (90%), they should have a greater role in chlamydia testing (79%) and require additional training or skills to manage testing and treatment of chlamydia (89%). The majority of the PNs wanted more involvement in chlamydia testing in their practices (79%) and to be involved in managing a recall/reminder system for chlamydia testing (75%). Over half indicated they would like involvement in discussing partner notification with patients who test positive for chlamydia (61%).

The most commonly identified barriers to increasing chlamydia testing were patients’ lack of chlamydia knowledge (69%) and time constraints during consultations (53%), followed closely by the lack of a formal chlamydia test recall/reminder system (46%) and lack of support for partner notification (46%). A third (33%) identified patient religion/ethnicity and a quarter (26%) lack of support for PNs as barriers, with less than a quarter identifying difficulty talking with clients about sexual health (21%) and cost of testing to client (21%). A very low proportion of nurses thought that the chance of patients getting a false positive result (10%) and concerns about over servicing (4%) were barriers to increasing chlamydia testing (see Table 3).

**Table 3:** Practice nurse opinions and perceived barriers to chlamydia testing

**Opinion statement**	**Agree/strongly agree n (%)**	**Disagree/strongly disagree n (%)**	**Neither agree/disagree n (%)**
PNs can conduct chlamydia testing in general practice	102 (89.5)	12 (4.4)	7 (6.1)
PNs should have a greater role in chlamydia testing	90 (78.9)	3 (2.6)	21 (18.4)
PNs require additional training/skills to manage chlamydia testing and treatment	100 (88.5)	6 (5.3)	7 (6.2)
I would like to be more involved with chlamydia testing in my practice	88 (78.6)	3 (2.7)	21 (18.7)
I would like to be involved with managing a recall/reminder system for chlamydia testing	84 (75)	7 (6.2)	21 (18.8)
I would like to be involved with discussing partner notification with patients who test positive for chlamydia	68 (60.7)	17 (15.2)	27 (24.1)
**Possible barriers to increasing chlamydia testing**	**Yes n (%)**	**No n (%)**	**Not sure n (%)**
Concerns about over servicing	5 (4.4)	81 (70.4)	29 (25.2)
Cost of testing to client	23 (20.5)	72 (64.3)	17 (15.2)
Time constraints	59 (52.7)	41 (36.6)	12 (10.7)
Difficulty talking with clients about sexual health	24 (21.4)	70 (62.5)	18 (16.1)
Lack of support for practice nurses	28 (25.5)	63 (57.3)	19 (17.2)
Chance of patients getting false positive	11 (9.8)	65 (58.1)	36 (32.1)
Patients lack of chlamydia knowledge	78 (69.1)	24 (21.2)	11 (9.7)
Religion/ethnicity of patient	37 (32.7)	51 (45.2)	25 (22.1)
Lack of formal chlamydia test recall/reminder system	52 (46.4)	42 (37.5)	18 (16.1)
Lack of support for partner notification	51 (46)	31 (27.9)	29 (26.1)

PN, practice nurse.

## Discussion

This survey identifies some important gaps in the chlamydia knowledge and self reported practices of GPs and PNs which may contribute to low chlamydia testing rates and suboptimal management of chlamydia in young people attending Australian general practice clinics. Knowledge of the age groups at greatest risk of chlamydia, especially in men, was poor in both professions. PNs demonstrated greater knowledge and more correct testing and retesting practices than GPs, but were less likely to collect the appropriate specimens for chlamydia testing. In addition, most of the GPs indicated that they were not prescribing the recommended antibiotic treatment for pregnant women with chlamydia. The study also demonstrated that although the majority of PNs were willing to become more involved in chlamydia testing and just over half in partner notification, they identified potential barriers to increasing testing including time constraints, lack of patient knowledge and lack of formal reminder systems to facilitate repeat testing.

The survey had a number of strengths. To our knowledge this is the first survey examining PN chlamydia knowledge and practice specifically and comparing the responses of PNs and GPs. Second, because of the direct questionnaire administration method and reminders the response rates were very high, comparable with the 85% response rate achieved by Mulvey et al. [20] and a vast improvement on more recent response rates achieved in other postal chlamydia knowledge and practice surveys in Australian general practice settings [17,19,21]. A similar system of sending reminders to non-responders was undertaken by Hocking et al. and achieved response rates of 60% [18].

A number of limitations should also be noted. About a quarter of clinicians did not return the questionnaire. The characteristics of these non responders are not available but it is possible they were less interested in sexual health and our study findings related to knowledge and practices may be overestimated. The ability to detect significant differences between professions was hampered by the lower sample size of PNs. Also the PNs who participated in the survey reflect nurses who were selected by their clinics to be involved in chlamydia testing if randomised to the intervention arm, whereas the majority of GPs were recruited. Thus the PNs may represent a more interested group of PNs, which may account for high levels of knowledge compared to GPs. The GPs are also unlikely to be representative of all Australian GPs, with most from rural towns where many overseas trained doctors are required to work for ten years before receiving access to Medicare benefits arrangements [28]. The result is an over- representation (41%) of overseas trained GPs in the sample compared with one-third in Australia GPs overall [29]. It should be noted however, that there were no significant differences in knowledge or correct practices between GPs trained overseas compared with those trained in Australia.

GPs reported they wouldn’t offer chlamydia testing opportunistically in a range of clinical presentations, including pap smears and pregnancy, which explains the low testing rate currently seen in general practice [11], and the low uptake of chlamydia testing in antenatal screening [30]. Testing only those with symptoms or reported risk will achieve a very low testing coverage, with the baseline prevalence survey of patients attending ACCEPt clinics finding only 5% presented for STI-related reasons [31]. Previous Australian and UK research examining GPs’ chlamydia knowledge and practice also found that GPs were less likely to offer testing during asymptomatic presentations, even those related to chlamydia testing, such as pap smears [19,21,32-34]. Conversely, the participating PNs in our survey thought that testing should be offered in *all* the asymptomatic non-sexual health scenarios presented, even in age groups outside of that recommended guidelines current at the time of the survey suggesting a greater commitment to opportunistic testing. This may be because nurses view sexual health as an integral part of holistic care and thus part of their role. PNs may also feel more comfortable discussing and offering chlamydia testing in unrelated consultations, compared with GPs, who may fear “offending” or “insulting” patients [16,35,36]. A reduced awareness of those age groups with the highest risk of chlamydia may also lead to missed opportunities for testing in the target age group and over testing in older age groups. It is very important that PNs are aware of the target age groups for chlamydia testing, as they are important providers of pap smears in Australian general practice and since 2006 have had the ability to undertake chlamydia testing at the time of cervical screening. Past initiatives linking chlamydia testing to pap smears have resulted in higher testing rates in older, lower risk age groups [37], and lower rates in younger women not eligible for cervical screening [38].

Inconsistencies in knowledge regarding recommended specimen collection suggest that respondents’ may be unfamiliar with guidelines for testing. Most identified acceptable specimens for asymptomatic patients and symptomatic female patients, but not for symptomatic heterosexual males and MSM. This could reflect lack of experience in STI testing for MSM due to the lower populations of MSM in rural areas or lack of awareness of patients’ sexuality due to not asking or gay men themselves not disclosing [39]. Research examining the comprehensiveness of STI testing in MSM shows that anal (and throat) swabs are the least common specimen collected [40,41].

By far, GPs had poorer knowledge about the need for chlamydia retesting and the appropriate time frame. This explains why in general practice only a quarter of young people are currently re-testing at 3 months following a positive chlamydia diagnosis and of those tested, only 7% are retesting in 12 months [42]. Recent data from England’s National Chlamydia Screening Programme (NCSP) revealed moderate annual repeat testing rates with 18% (overall NCSP) to 26% (GUM clinics) of young people re-tested within one year, and a higher proportion of positive retests in those who tested positive at baseline [10]. Lack of clinician awareness or inconsistencies in the guidelines recommendations on the timing of retesting may explain Australian GPs poor knowledge in this area. RACGP guidelines advise repeat testing from 3-12 months following chlamydia infection, compared with other STI management guidelines recommending 3 months [9,26]. However, within our sample, over half of the GPs (63%) who identified the need for a repeat test following chlamydia diagnosis thought that this test should be performed before 12 weeks, with just under half (44%) choosing between 1 to 5 weeks, a timeframe in which retesting may result in a false positive result due to the presence of non-viable chlamydial DNA [43]. GPs self reported treatment practices are also suggestive of unfamiliarity with current guidelines. Whilst the majority indicated that they would usually prescribe azithromycin for men or non-pregnant women, under half chose azithromycin for the treatment of chlamydia infection in pregnant women. Almost the same proportion would use amoxicillin or erythromycin, which despite being effective in treating chlamydia infection, are associated with more adverse gastro-intestinal side effects and poorer adherence than azithromycin [44].

It is encouraging to note that not only do PNs in this survey demonstrate good baseline chlamydia knowledge but they are also willing to undertake a role in all aspects of chlamydia management, including partner notification and management of recall systems for chlamydia retesting, whilst also identifying the need to undergo additional training to do so. The perceived barriers to increasing chlamydia testing were consistent with those previously identified by Australian GPs in a study by Hocking et al. [35] and GPs and PNs in the UK [15,16,34,36].

## Conclusions

In conclusion, chlamydia infection and re-infection are important health issues for young people. General practice is ideally placed to implement a successful chlamydia screening programme that may have the potential, through increasing testing, to impact on the burden of chlamydia in this population. Despite this, testing rates in general practice are poor and as this survey reveals, important gaps in chlamydia knowledge and practice and barriers to testing exist amongst clinicians. To fully support clinicians in achieving increased testing rates, these gaps and barriers must be addressed. The multifaceted ACCEPt intervention, which is tailored to each participating clinic, is appropriately designed and targeted to achieve this. Finally, PNs, with their demonstrated levels of chlamydia knowledge and willingness to offer testing opportunistically have the potential to make a significant contribution to chlamydia testing in general practice and therefore strategies to increase their involvement must be explored.

## Competing interests

The authors declare that they have no competing interests

## Authors’ contributions

RL devised the research question, analysed the data and drafted the manuscript. JH, RG, MTS contributed to the study design, supervised data analysis and writing of the manuscript. AY, AW, AV contributed to development of study instruments, administered study instruments and contributed to writing of the manuscript. ML contributed to the study design, supervised data analysis and writing of the manuscript. CF, BD, JK contributed to the study design and writing of the manuscript. All authors read and approved the final manuscript.

## Pre-publication history

The pre-publication history for this paper can be accessed here:

http://www.biomedcentral.com/1471-2296/14/169/prepub
